# New Concepts in Diagnostics for Invasive Mycoses: Non-Culture-Based Methodologies

**DOI:** 10.3390/jof5010009

**Published:** 2019-01-17

**Authors:** Thomas F. Patterson, J. Peter Donnelly

**Affiliations:** Division of Infectious Diseases, San Antonio Center for Medical Mycology, The University of Texas Health Science Center at San Antonio and the South Texas Veterans Health Care System, 7703 Floyd Curl Drive-MSC 7881, San Antonio, TX 78229-3900, USA; p.donnelly@usa.net

**Keywords:** invasive fungal infection, non-culture-based diagnostics, aspergillosis, candidiasis, *Aspergillus* PCR, galactomannan, lateral flow, beta-d-glucan, T2 *Candida*

## Abstract

Non-culture-based diagnostics have been developed to help establish an early diagnosis of invasive fungal infection. Studies have shown that these tests can significantly impact the diagnosis of infection in high risk patients. *Aspergillus* galactomannan EIA testing is well-recognized as an important adjunct to the diagnosis of invasive aspergillosis and can be detected in serum, bronchoalveolar lavage and other fluids. Galactomannan testing used along with PCR testing has been shown to be effective when integrated into care paths for high risk patients for both diagnoses and as a surrogate marker for outcome when used in serial testing. Beta-d-glucan assays are non-specific for several fungal genera including *Aspergillus* and *Candida* and in high risk patients have been an important tool to augment the diagnosis. Lateral flow technology using monoclonal antibodies to *Aspergillus* are available that allow rapid testing of clinical samples. While standard PCR for *Candida* remains investigational, T2 magnetic resonance allows for the rapid diagnosis of *Candida* species from blood cultures. *Aspergillus* PCR has been extensively validated with standardized approaches established for these methods and will be included in the diagnostic criteria in the revised European Organization for Research and Treatment of Cancer/Mycoses Study Group (EORTC-MSG) definitions. Finally, these non-culture-based tests can be used in combination to significantly increase the detection of invasive mycoses with the ultimate aim of establishing an early diagnosis of infection.

## 1. Introduction

Invasive fungal infections remain a significant cause of morbidity and mortality in immunocompromised patients. The diagnosis of these infections is delayed due to lack of positive cultures from blood or from tissues, which require invasive procedures to obtain and are often difficult to perform in these critically ill patients. Non-culture-based diagnostics have been developed to help establish an early diagnosis of infection with the aim of allowing prompt initiation of antifungal therapy and improving patient outcomes.

Non-culture-based diagnostics have been developed for both *Aspergillus* and *Candida* along with other opportunistic fungal pathogens [1]. These assays have been largely focused on *Aspergillus* due to its prominence as the most common mold in immunocompromised hosts and for *Candida,* to augment diagnosis in the setting of negative or delayed positive blood cultures [2]. Assays are being developed for opportunistic pathogens including mucorales but are less widely available in clinical settings [3,4]. Additionally, for endemic fungi including *Coccidioides, Histoplasma,* and *Blastomyces* as well as *Cryptococcus*, non-culture-based methods for diagnosis are available but beyond the scope of this review.

Non-culture-based tests include galactomannan, which can be used in serum, bronchoalveolar lavage fluid and other samples; beta-d-glucan, a non-specific assay for *Aspergillus, Candida,* and other mycoses; lateral flow technology using an *Aspergillus* monoclonal antibody; and others including *Candida* PCR and T2 magnetic resonance. *Aspergillus* PCR has been extensively validated for standardized methodologies and is now included in the recent EORTC/MSG definition updates. In this review, the data supporting the use of clinically available non-culture-based methods for *Aspergillus* and *Candida* will be discussed and their utility alone and in combination will be summarized.

## 2. Risk Factors and Impact of Diagnostics

When approaching the use of these assays in the clinical setting, it is important to recognize the risk factors associated with invasive fungal infection, in order to improve the utility of their performance. The risk factors for invasive fungal infections have been extensively evaluated, as they significantly impact the incidence of invasive fungal infections and thus the performance of diagnostic assays. Herbrecht and colleagues outlined host factors for ‘high risk patients’, including those with allogeneic stem cell transplants, acute myelogenous leukemia/myelodysplastic syndrome, chronic granulomatous disease and others; those at ‘intermediate’ risk, including solid organ transplant recipients, other haematological malignancies, uncontrolled HIV infection, and others; while ‘low risk’ includes patients with autologous stem cell transplants, kidney transplant, solid tumors and others [5]. Additional risk factors influence the host condition, including innate immune defects; underlying conditions (neutropenia, graft vs. host disease, corticosteroid use, other biological agents, chemotherapy, etc); environmental factors and exposures; and other co-morbidities (diabetes, respiratory diseases and others) [5].

Fleming and colleagues established a risk stratification for patients with hematological malignancies. High risk patients are those with >10% incidence of invasive fungal disease, that is, patients with prolonged neutropenia (<0.1 × 10^9^/L for > 3 weeks or <0.5 × 10^9^/L for >5 weeks), unrelated, mismatched or cord blood donor SCT, graft vs. host disease (GVHD), high doses of corticosteroids, certain chemotherapeutic agents (high-dose cytarabine, fludarabine, alemtuzumab, and others), and certain hematological malignancies (acute myelogenous leukemia (AML) and acute lymphocytic leukemia (ALL)) [6]. An intermediate risk group with an incidence of invasive fungal disease of around 10% includes those with less profound neutropenia (0.1–0.5 × 10^9^/L for 3–5 weeks or 0.1–0.5 × 10^9^/L for <3 weeks with lymphopenia), while low risk patients (~2% incidence of invasive fungal disease) would include autologous SCT and lymphoma [6]. Clearly, these patients with hematological malignancies have significant differences in risk for fungal infection and it becomes critically important to consider these differences when interpreting the clinical utility of these non-culture-based diagnostic tests, based on the prior probability of disease.

It is also critical to recognize the impact that diagnostic tests can have on underestimates of infection and the impact that diagnosis has on outcomes. Ceesay and colleagues evaluated a series of 203 patients with hematological malignancies using a strict diagnostic algorithm including a pre-treatment computed tomography of the chest, twice weekly serum galactomannan, and beta-d-glucan with suspicion of infection and tissue for diagnosis [7]. The series showed that the incidence of established infection rose from 10.5% with galactomannan alone to almost 20% with a combination of galactomannan and beta-d-glucan, and was 21.1% when all tests were combined. Furthermore, at 45%, the survival of those with proven/probable infection was significantly lower than those with possible disease, at 66%. The survival rate was 87% for those without infection (*p* < 0.001), supporting the importance of using these tools to establish a diagnosis of invasive fungal disease.

## 3. Galactomannan

The detection of galactomannan by EIA is a well-established and extensively studied method for the diagnosis of invasive aspergillosis [2,8,9]. Monoclonal antibody EB-A2 is used in a double sandwich ELISA to detect an antigenic side chain of β-1,5-galactofuronosyl with a linear core of mannan with α1,2 and α1,6 linkages [10]. Early studies by Maertens and others prior to the availability of anti-mold prophylaxis showed a sensitivity and specificity of 89% and 98%, respectively [11]. Subsequently, other studies showed more limited sensitivity (43–70%) but with specificity of 70–93% and studies confirmed the validation of a galactomannan index (GMI) of 0.5 as the threshold for positivity [12,13]. It was also appreciated that there were several sources for false-positive results including weakly positive samples, cross-reactivity with other fungi and antibiotics (such as pipercillin-tazobactam, which has now been resolved) [14]. Other sources of false-positives such as dietary reactivity, laboratory contamination or fluids for bronchoalveolar lavage (such as plasmalyte which also appears to have resolved) may continue [14,15,16,17,18,19]. A number of other factors may affect the performance of the galactomannan assay, including biological factors (such as site of infection, *Aspergillus* species, prior use of antifungals, renal clearance, hepatic metabolism, underlying condition, storage of the samples, and others) and epidemiological factors (such as the prevalence of disease, sampling strategies and definitions for positive results) [17]. An important study by Duarte and colleagues showed the dramatic impact of antifungal prophylaxis on the strategy of serial sampling [20]. In this study the positive predictive value dropped to 11.8%, but was still useful with a positive predictive value of 89.6% when used for the diagnosis of invasive fungal disease on suspicion of disease [20].

Galactomannan testing in bronchoalveolar (BAL) lavage fluid is very useful for establishing a diagnosis of infection. This method is more sensitive than cytology, culture, transbronchial biopsy or serum galactomannan testing [21]. Galactomannan detection increased the sensitivity from serum from 47% to 85% from BAL, with a positive predictive value approaching 100% and with a BAL GMI of <0.5, indicating that it is useful to exclude the diagnosis in high risk patients with hematological malignancies [22,23]. On the other hand, in solid organ transplant patients, false positive results were more likely in lung transplant patients and in those colonized with *Aspergillus* [24]. The utility of galactomannan has been shown to improve in combination with PCR. Reinwald and colleagues showed that positivity from BAL of both galactomannan (at a GMI of >0.5) with positive PCR results highly supported the diagnosis [25]. Notably, the consensus regarding the cutoff value for a positive BAL galactomannan is still lacking, as performance will also vary in different patient settings (i.e., hematological malignancy, solid organ transplantation, intensive care units, etc.) so that a higher cutoff threshold (GMI > 1.0) may correlate with better diagnostic utility [26,27].

Galactomannan along with PCR was evaluated in a diagnostic vs. empirical therapy approach by Morrissey and colleagues [28]. In this study, empirical antifungal therapy was reduced from 32% in the standard diagnosis group to 15% in the biomarker diagnosis group, even though the rate of proven/probable disease was increased from 1% to 15% [28]. Mortality was 15% in the standard diagnosis group and 10% in the biomarker group [28]. In this study, 10/39 (26%) of the patients receiving empirical antifungal therapy would have been diagnosed with invasive aspergillosis a median of 4 days earlier and 5/6 (83%) of those who died from invasive aspergillosis would have been diagnosed a median of 7 days earlier.

Finally, serial galactomannan measurements can also be used for the assessment of outcomes. Chai and colleagues showed that a GMI reduction of >35% between baseline and week 1 predicted a satisfactory response in patients enrolled in the Global Voriconazole Aspergillosis trial [29]. Poorer responses occurred with increasing GMI after 2 weeks. Similarly, outcomes are better in patients who become GMI negative during their course of treatment [9,30].

## 4. Beta-d-Glucan

Testing with (1-3)-β-d-glucan activates *Limulus* amebocyte lysate through factor G initiation of the complement cascade [31]. The output can be measured using a chromogenic substrate (Fungitell (Associates of Cape Cod, Falmouth, MA) and others or by turbidity after gel clot (Wako Pure Chemical Industries, Osaka, Japan) [31]. These assays detect a number of important fungal genera including *Aspergillus, Candida, Trichosporon, Fusarium,* and *Exerohilum* but not mucorales or cryptococcosis [32]. Early studies showed the value of the assay in candidemia and in invasive fungal disease in patients with acute leukemia, which allowed regulatory clearance as well as inclusion in the European Organization for Research and Treatment of Cancer/Mycoses Study Group (EORTC-MSG) definitions for fungal infection [33,34].

More recent studies in invasive candidiasis have evaluated the role of beta-d-glucan with *Candida,* real-time PCR and blood cultures [35]. In this study, both PCR and beta-d-glucan were more sensitive for deep seated *Candida* infection: 88% and 62%, respectively, vs. 17% for blood cultures. Beta-d-glucan was shown to anticipate the diagnosis of blood culture-negative intraabdominal candidiasis and may be an important adjunct to that diagnosis [36].

Beta-d-glucan testing has also shown utility in specific clinical settings. In pneumocystis pneumonia, beta-d-glucan levels are frequently extremely elevated (>500 pg/mL) so that in a likely clinical setting more invasive testing might be obviated [37]. In addition, during an outbreak of fungal meningitis due to contaminated steroids, it was recognized that *Exserohilum* spp. (the etiological cause of the outbreak) produces high levels of beta-d-glucan [38]. It was subsequently shown that beta-d-glucan is highly sensitive for the diagnosis and is correlated with response to therapy [38].

## 5. Lateral Flow Technology in Invasive Aspergillosis

Lateral flow assays for invasive aspergillosis offer the potential for a rapid diagnosis, ease of performance and point of care use. A murine monoclonal antibody, JF4, binds to an extracellular glycoprotein antigen secreted during the growth of *Aspergillus* and distinguishes between hyphae and conidia [39]. Earlier and more consistent detection compared to beta-d-glucan or galactomannan was seen in pre-clinical models [39,40,41]. A prototype lateral flow device (LFD) was evaluated in high risk hematological malignancy patients and was shown to improve the diagnostic yield, especially when combined with galactomannan and PCR [42,43,44]. In BAL samples this assay had an overall sensitivity of 73% and a specificity of 90% [45,46]. A recent European conformity (CE)-marked LFD (OLM Diagnostics, Newcastle-on-Tyne, UK) showed similar sensitivity to the prototype device of 71%, but with an improved sensitivity of 100% [46]. Another lateral flow assay for *Aspergillus* (IMMY, Norman, OK, USA) was compared in a small study to the LFD in BAL fluid and showed similar sensitivity and specificities of 89% and 88% [47].

## 6. T2 Magnetic Resonance

While real-time PCR for *Candida* remains investigational, the use of T2 magnetic resonance using nanoparticles has been cleared for clinical use (T2 *Candida,* T2 Biosystems, Lexington, MA, USA) [48,49,50]. The assay requires a dedicated instrument and detects *Candida* species directly from blood samples, but unlike blood cultures it does not require viable organisms [51]. The assay detects five major *Candida* species that are grouped based on typical susceptibility patterns. Using spiked blood samples, it was shown that this method could detect *C. albicans/C. tropicalis, C. parapsilosis,* and *C. krusei/C. glabrata* at a sensitivity of 91.1% with a time to positivity of 4.4 hrs and a limit of detection of 1–3 CFU/mL [52]. In a follow-up study of patients with candidemia, follow-up blood cultures were compared with the T2 *Candida* assay and showed T2 *Candida* positivity in 45% of the follow-up blood cultures compared to 24% with standard culture techniques. These results suggest that this method could be used to detect candidemia in patients who are receiving empirical antifungal therapy and could be very useful in allowing empirical therapy to be discontinued in patients with negative results [51]. In addition, T2 shortened the time to positivity to <3 h and identified a bloodstream infection not detected by blood cultures, while retaining sensitivity during antifungal therapy.

## *7. Aspergillus* PCR Development and Standardization

PCR for *Aspergillus* species has been evaluated for more than 25 years to fulfil the mycological criteria for the diagnosis of invasive fungal disease [53]. The EORTC/MSG clinical definitions were published in 2002 and revised in 2008 and provided important guidelines for criteria of proven, probable and possible invasive fungal disease, in order to facilitate clinical research including drug trials, epidemiology, and diagnostic tests [54,55]. These were not intended as a guide to clinical practice, but the elements of the definitions have been widely used as an adjunct to clinical management. The 2008 definitions defined proven disease when fungi are detected in specimens from a sterile body site or in a biopsy. In contrast, possible and probable disease both require a host factor and a clinical feature. Possible disease is assigned in the absence of any mycological criteria. Probable disease is assigned when the mycological criteria are met by direct mycology–cytology, direct microscopy or culture, or indirectly, by the detection of galactomannan and beta-d-glucan, but not PCR, due to the lack of standardization and validation [55]. Subsequently, the European *Aspergillus* PCR Initiative (EAPCRI) was established to develop a standard for *Aspergillus* PCR methodology so that PCR could be incorporated into future consensus definitions for invasive fungal disease [56].

Initial efforts included a systemic review published in 2009 of more than 10,000 blood, serum or plasma samples from 1618 patients at risk for *Aspergillus* and concluded at that time that two positive tests were required to confirm the diagnosis due to the specificity of these tests and a single PCR-negative result was sufficient to exclude a diagnosis of invasive aspergillosis [57]. However, it was also noted that there was a lack of homogeneity of the PCR methods so that subsequent collaboration aimed at a formal validation of that process.

*Aspergillus* standardization was evaluated through a collaboration of 21 European medical centers [56]. While specific protocols were not developed, *compliant* and *non-compliant* centers were noted. Twelve centers used 10 different DNA extraction protocols and nine different PCR amplification procedures that were compliant. Nine centers used seven extraction protocols and seven different PCR amplification procedures that were noncompliant. The sensitivity, specificity, and diagnostic odds ratio (DOR) of compliant centers were 88.7%, 91.6%, and 119.9, respectively, for non-compliant centers these were and 57.7%, 77.2%, and 8.9, respectively. These results were highly statistically significant for sensitivity (*p* = 0.008) and DOR (*p* = 0.006). The factors with the most influence on PCR included compliance with the extraction method, bead beating of the sample, and use of an internal control PCR. This led to recommendations for the whole blood PCR of >3 mL of blood, bead-beating to lyse fungal cells, a real time PCR platform with multi-copy targets with specific probes, internal control PCR and others [56]. Subsequent work showed that most of the *Aspergillus* protocols used to test serum generated satisfactory analytical performance and that the testing serum required less standardization [58]. Additional evaluation evaluated plasma vs. serum and showed improved sensitivity with plasma with PCR positivity occurring earlier, while maintaining methodological simplicity [59].

An extensive Cochrane study was performed for the diagnosis of invasive aspergillosis in immunocompromised people [60]. The studies included were those that compared the results of blood PCR tests with reference to the EORTC/MSG standard and included false positive, true positive, false negative and true negative results and that evaluated the test(s) prospectively in cohorts from patients at high risk for invasive aspergillosis. Overall, 1672 records were identified and 155 were screened to eventually include 18 studies for the meta-analysis [60]. For one single PCR specimen a sensitivity of 80.5% and a specificity of 78.5 were reported in 17 studies. For two or more PCR specimens the sensitivity decreased to 57.9% and the specificity increased to 96.2%. The authors concluded that PCR shows moderate diagnostic accuracy when used for screening and has a high negative predictive value (NPV) that allows the diagnosis of invasive aspergillosis to be ruled out. A poor positive predictive value (PPV) when the prevalence of disease is low, limits the ability to rule in a diagnosis. Since other non-culture-based methods (such as galactomannan) detect different aspects of the disease, combinations of both together are likely to be more useful. PCR used in combination with galactomannan has been shown to improve the detection of invasive aspergillosis before detection by CT findings in high risk hematological patients [61] and to improve the sensitivity and specificity of testing [62]. In a randomized controlled trial, a combined monitoring strategy based on serum galactomannan and *Aspergillus* PCR was associated with an earlier diagnosis of invasive aspergillosis [63]. Robust recommendations for plasma and serum are available that establish a ‘standard for PCR’ but not a ‘standard method for PCR’ so that *Aspergillus* PCR will be included in the revised EORTC/MSG consensus definitions [64].

## 8. Clinical Utility and Summary

The importance of assessing risk and using non-culture-based diagnostics for invasive fungal disease is clear. Several methods have been evaluated and validated for clinical use including galactomannan, beta-d-glucan, lateral flow technology, T2 magnetic resonance, PCR and others. Non-culture-based biomarkers provide more reliable negative than positive predictive values. When the prevalence of disease is higher than 15%, negative test results exclude the diagnosis, while positive test results include the diagnosis. Clinicians and laboratories need to consider when a test is being requested for screening (when a patient is at risk for invasive fungal disease) as opposed to diagnosis (in which there is a high clinical suspicion of an invasive fungal disease), which will have a substantially higher pre-test probability. Finally, combinations of these tests may provide the greatest benefit in establishing a diagnosis of invasive fungal disease.
